# The Relationship among Internet Addiction, Moral Potency, Mindfulness, and Psychological Capital

**DOI:** 10.3390/ejihpe14060115

**Published:** 2024-06-16

**Authors:** Girum Tareke Zewude, Tun Zaw Oo, Gabriella Józsa, Krisztián Józsa

**Affiliations:** 1 Department of Psychology, Wollo University, Dessie 1145, Ethiopia; girum.tareke@wu.edu.et; 2 Institute of Education, Hungarian University of Agriculture and Life Sciences, 7400 Kaposvár, Hungary; oo.tun.zaw@uni-mate.hu; 3 MTA-MATE Early Childhood Research Group, Hungarian University of Agriculture and Life Sciences, 7400 Kaposvár, Hungary; 4 Faculty of Pedagogy, Károli Gáspár University of the Reformed Church, 2750 Nagykőrös, Hungary; 5 Institute of Education, University of Szeged, 6722 Szeged, Hungary

**Keywords:** internet addiction, moral potency, mindfulness, psychological capital

## Abstract

This research aimed to contribute to the literature on internet addiction (IA) and moral development among university students. Moral potency (MP) encompasses the interconnected dimensions of moral courage, moral ownership, and moral efficacy. Studies on the relationships between students’ problematic behaviors (e.g., IA) and cognitive processes like MP, mindfulness (MI), and psychological capital (PsyCap) are scarce in educational research. Therefore, this study investigated the relationships among IA, MP, MI, and PsyCap in university students. This study included 868 undergraduate students from a state university in Ethiopia, with 526 male students (60.6%) and 342 female students (39.4%). Participants’ ages ranged from 21 to 29 years, with a mean age of 22.31 and a standard deviation of 4.03. The findings indicated that IA was negatively correlated with MI, PsyCap, and MP. Both MI and PsyCap showed positive correlations with MP. Importantly, this study revealed that IA had a direct and negative impact on MI, PsyCap, and MP. Further, MI and PsyCap partially mediated and fully mediated the relationship between IA and MP. These findings suggest that cultivating MI and positive PsyCap among university students could be an important strategy to reduce the risks of IA and enhance their moral development. This study contributes to the limited research on the complex relationships between technology use, psychological resources, and moral functioning in emerging adulthood.

## 1. Introduction

The rapid expansion of internet usage and the increasing prevalence of social media platforms have raised concerns about their impact on individuals’ moral values in the digital era [1,2]. Internet addiction (IA), also known as problematic internet use, has garnered significant attention from researchers and experts across various disciplines [2,3]. It refers to excessive or compulsive internet use that leads to significant impairment in different aspects of individuals’ lives over an extended period [3]. A particular interest is the relationship between IA and moral development among adolescents [4,5,6,7]. Researchers have explored how IA can potentially impact adolescents’ moral potency (MP), which encompasses their ability to achieve moral purpose, exhibit moral courage in the face of adversity, and persevere through challenges [8].

Moral potency (MP) is a psychological state influenced by the context in which a student operates, and it is more open to change. Some students may exhibit higher levels of MP to decrease IA when faced with specific contexts. MP helps students understand that it is their place to act (moral ownership), believe in their ability to succeed in those actions (moral efficacy), and overcome fears to persevere and see those actions through to resolution (moral courage) [8]. Consequently, IA is a multifaceted phenomenon that has been investigated from diverse perspectives related to adolescents’ mental health and MP. For example, research has explored the relationship between IA, mindfulness (MI), psychological capital (PsyCap), and MP. Researchers [9] found a connection between IA and psychological health, suggesting that IA can have implications for moral values and ethical considerations relevant to psychological well-being.

The interaction between behavior and mind is a topic of continuing controversy in psychology [10]. There are several studies supporting epistemological points of view that behavioral patterns can affect cognition (e.g., IA affects moral potency). For example, a study [11] also examined the association among moral potency, IA, and mental health, finding that internet addiction predicted moral potency and mental health. Similarly, Zewude and colleagues [12] also explored the relationship between psychological capital and internet addiction, finding that excessive internet use was associated with lower PsyCap and poorer mental health. On the contrary, there is also evidence that understanding an individual’s intentional and belief states relies on exteroceptive, proprioceptive, and interoceptive signals, which inform intentions and beliefs that guide behavior [13]. In this sense, there is clear evidence supporting the interdependence between behavior and thought. A theory of behavior research setting has also shown the interdependence between behavior and thought, emphasizing the cultural nature of the ecology of human psychology [14]. Moreover, researchers [15] investigated the relationship between IA, mindfulness, and mental health outcomes, highlighting the inverse association between IA and mindfulness, as well as the positive impact of mindfulness on mental health.

The multifaceted nature of IA has prompted investigations into various aspects, particularly adolescents’ moral potency. As moral potency helps students understand their behavior, their ability, and their courage to overcome challenges [12], it is important to nurture students to have good moral potency in life. In the current age, most students have behavioral problems of IA, which have several relationships with their mental health, moral potency, and other developmental aspects. However, studies focusing on the relationships of students’ behaviors (e.g., IA) and other important cognitive processes, such as MP, MI, and PsyCap, are scarce in the realm of educational research. Therefore, this study aims to investigate the relationships among students’ internet addiction (IA), moral potency (MP), mindfulness (MI), and PsyCap.

## 2. Literature Review

This study aimed to investigate the relationships among several terms of behavior and thoughts, such as internet addiction (IA), moral potency (MP), mindfulness (MI), and psychological capital (PsyCap). IA has been viewed as a problematic behavior that can have negative impacts on students’ thoughts in MP, MI, and PsyCap. Accordingly, the meaning and nature of IA are defined, as well as its related factors such as PsyCap, MI, and MP. Subsequently, their related findings are presented coherently and the hypothesized model for current research was formulated.

### 2.1. Internet Addiction (IA) and Psychological Capital (PsyCap)

IA refers to the excessive and compulsive use of the internet that negatively impacts various aspects of a person’s life, including physical, psychological, social, and professional domains [16]. It is characterized by an inability to regulate or limit internet use, a preoccupation with online activities, withdrawal symptoms when offline, and detrimental effects on relationships, work, or school performance, as well as overall mental well-being [11].

On the other hand, PsyCap refers to a person’s favorable psychological state and resourcefulness, encompassing four key components that can potentially have a direct or mediating role in relation to MP. These components include (1) hope: this involves maintaining positive expectations and motivation to pursue goals, even in the face of obstacles; (2) self-efficacy: this refers to the belief in one’s ability to successfully accomplish tasks and overcome challenges; (3) optimism: this entails having a positive outlook and expecting favorable outcomes; and (4) resilience: this refers to the capacity to recover and adapt in the face of difficulties [17].

Research has suggested that PsyCap, including its components of hope, self-efficacy, resilience, and optimism, can play a protective role against IA and increased social adaptation [18,19]. In addition, PsyCap and MI played a mediator role in smartphone addiction and mental well-being and increased life satisfaction [19,20]. A higher level of PsyCap and MI can empower individuals to control and regulate their internet use, establish boundaries, engage in healthier online behaviors, seek help, make positive changes to overcome IA, and develop strategies to manage their internet use more effectively and develop a higher level of MP and mental health [12,20,21]. The author also found that excessive internet use can have negative effects on PsyCap, MI, and life satisfaction. Individuals addicted to the internet may experience decreased self-efficacy as they struggle to control their online behavior [19,21]. Furthermore, excessive internet use can lead to a loss of hope and optimism as individuals become socially isolated and neglect real-life relationships and goals. Additionally, IA can undermine resilience, making it challenging for individuals to cope with the negative consequences of their excessive internet use.

To sum up, IA has detrimental effects on various aspects of a person’s life, while PsyCap, including its components of hope, self-efficacy, resilience, and optimism, can serve as a protective factor against IA [22]. The authors also added that excessive internet use can negatively impact PsyCap, leading to decreased self-efficacy, diminished hope and optimism, and compromised resilience.

### 2.2. Internet Addiction (IA), Mindfulness (MI), and Moral Potency (MP)

University students are widely acknowledged as a vulnerable group prone to psychological health and moral decline, mainly attributed to their maladaptive health behaviors and the demanding nature of their academic programs, and involved in unethical behaviors [20]. However, one area of study that has received less emphasis is IA.

In today’s world, IA has had a detrimental impact on people’s psychological and mental well-being and moral virtue, with adolescents being particularly vulnerable [12,23,24]. Several research studies [6,11] have identified MI and positive PsyCap as the most powerful positive psychological resources for safeguarding ego strength and reducing levels of IA. These constructs are crucial for designing interventions and prevention strategies. Furthermore, they play a vital role in supporting an individual’s overall well-being, including students’ MP and functioning, especially when faced with severe levels of IA [6,11].

MI, defined as the practice of being fully present and engaged in the present moment without judgment [25], has been extensively studied for its numerous benefits. These include reducing depression and promoting well-being [15], buffering against IA [11], improving job performance and reducing turnover intention [26], enhancing the concept of self and reducing stress levels, and fostering moral cognition [27].

For instance, researchers [11] conducted a study exploring the potential benefits of MI interventions in reducing problematic internet use. They found that MI training was associated with a decrease in IA symptoms. Another survey by Sheykhangafshe et al. [28] revealed that IA, MI, and resilience as positive psychological key constructs significantly predicted students’ mental health. A study [6] also reported negative correlations between MI and stress and IA, along with a positive correlation with self-control. Notably, individuals who regularly practice MI have been described as better equipped to overcome adversity, particularly in challenging conditions faced by students [11,27]. By emphasizing the importance of MI and psychological resources, researchers aim to develop interventions and preventive measures to address the growing concern of IA among the MP of university students.

### 2.3. Internet Addiction (IA), Psychological Capital (PsyCap), and Moral Potency (MP)

There has been relatively little research conducted on the connection between internet addiction (IA) and psychological capital (PsyCap) and moral potency (MP). Internet addiction refers to the excessive and compulsive use of the internet, leading to adverse effects on different aspects of an individual’s life, including their mental health and general well-being [1]. On the other hand, psychological capital pertains to a person’s positive psychological state characterized by hope, efficacy, resilience, and optimism [17]. While there is limited research specifically examining the relationship between IA and PsyCap, there is evidence to suggest that excessive internet use can have a negative impact on PsyCap [21]. Earlier studies have found a correlation between internet addiction (IA) and heightened indications of depression, anxiety, and diminished self-esteem, all of which can have a detrimental impact on psychological capital (PsyCap) [21]. Individuals affected by IA may exhibit lower levels of resilience, optimism, hope, and self-efficacy. However, additional research is required to establish a direct and definitive relationship between these variables. Furthermore, IA negatively predicts MP. The literature states that MP is essential for ethical behavior and can influence various aspects of an individual’s life, including their relationships, decision making, and overall well-being [29,30]. Individuals with higher MP may be less prone to excessive internet use due to their strong sense of values and self-regulation [21]. However, problematic internet use can potentially impact moral decision making and behavior [31]. In addition, excessive internet use can lead to a decrease in face-to-face social interactions and a blurring of online and offline identities [32]. This may result in reduced opportunities for individuals to practice and develop moral values and ethical behavior.

It is crucial to acknowledge that the research exploring the interplay between internet addiction (IA), psychological capital (PsyCap), and moral potency (MP) is still in its preliminary phases. Consequently, there is a pressing requirement for additional investigations to yield more substantial and reliable findings [8,33,34,35,36]. The relationship among these constructs is anticipated to be intricate, influenced by diverse individual and contextual factors. Furthermore, the direction of causality remains uncertain, with the possibility that IA can both influence and be influenced by PsyCap and MP. Overall, while there is limited research specifically linking IA to PsyCap and MP, it is reasonable to assume that excessive internet use can have a negative impact on psychological well-being and potentially affect an individual’s moral decision making.

### 2.4. Findings from the Review of Internet Addition (IA) and Its Several Relations

Other studies have also examined the relationship between social media overuse, moral values, psychological well-being, and moral disengagement [34,35,36]. These studies highlight the complex relationship between IA, MI, PsyCap, MP, and mental health. They suggest that excessive internet use and social media platforms can have detrimental effects on individuals’ moral values, psychological well-being, and overall mental health. Consequently, it is crucial to continue researching and raising awareness about these issues to develop effective strategies for prevention and intervention.

Emerging research has shed light on the interplay between problematic social media use, moral faculties, psychological resources, and mental health. Studies have found that excessive engagement with social media and internet addiction (IA) are linked to diminished moral values, reduced psychological capital (PsyCap), and poorer overall mental health [12,34]. Further investigations have specifically examined the relationship between IA, PsyCap, and mental health outcomes. This work indicates that higher levels of IA are associated with a lower PsyCap and more compromised mental wellbeing [36]. Researchers have also explored how IA relates to moral values, moral identity (MI), and psychological well-being more broadly. Their findings suggest that greater IA corresponds with lower moral values, diminished MI, and poorer psychological wellness [6,20,37]. Finally, studies have examined the connections between social networking site (SNS) addiction, IA, moral disengagement, and mental health problems. This research reveals that both SNS addiction and elevated IA are linked to heightened moral disengagement and worse mental health outcomes [36]. Taken together, these studies underscore the concerning links between problematic digital technology use, moral capacities, psychological resources, and overall mental health. This evidence underscores the need to address excessive or addictive social media and internet use as part of promoting individual and societal wellbeing.

Furthermore, several studies [8,12,19,20,23] have examined college and university students’ mobile phone addiction, MI, MP, PsyCap, life satisfaction, and mental health. All these studies showed that IA affected the mental health and moral development of college students. These studies recommended that positive PsyCap and MI played a potential role in mediating these relationships. PsyCap and MI are both constructs drawn from *the field of positive psychology*, but they have distinct objectives, focuses, and measurement approaches [17]. PsyCap encompasses positive psychological resources such as hope, resilience, optimism, and self-efficacy, which are leveraged to mitigate the adverse effects of stress, challenges, and negative circumstances. The aim of PsyCap is to enhance individuals’ capacity to cope, adapt, and thrive in the face of difficulties [17]. In contrast, MI refers to the practice of being present and fully engaged in the current moment, without judgment or distraction [15]. MI involves cultivating awareness and acceptance of one’s thoughts, feelings, and sensations as they arise, without getting caught up in them. The specific components of MI include acting with awareness, describing, observing, non-judging, and non-reactivity. While both PsyCap and MI originate from positive psychology, PsyCap emphasizes positive psychological resources as protective factors, whereas MI focuses on cultivating a state of present-moment awareness and non-judgment [15,17]. By highlighting these conceptual differences, we aim to clarify how PsyCap and MI contribute to the overall model and provide distinct pathways for understanding and addressing internet problematic use and play a mediating role between IA and MP.

The prevalence of IA among university students varies across different batches or years of study. For example, a study involving undergraduate students at Sevastopol State University found differences in valuable and semantic orientations among students with IA, based on their year of study or batch [38]. Another study at Erciyes University observed an increase in internet use over the years, with a higher proportion of students reporting addiction symptoms as they progressed through the university [39]. A study in Jordan found that university year level or batch was a significant correlate of IA among students [40]. The level of IA in university students was assessed, and it was found that there is no significant gender difference in IA [41,42]. However, some studies indicated that males showed significantly higher scores in IA than females [43,44,45].

While studies have been conducted on various aspects related to IA, MI, PsyCap, and MP, there are still significant research gaps that need to be addressed. Four major research gaps can be identified in these areas.

Firstly, associational research of behavior and thoughts (e.g., the relationships among IA, MI, PsyCap, and MP) is scarce in the field of education in Ethiopia and European contexts. The overuse of the internet among adolescents, not only in Ethiopia but also in many countries worldwide, can lead to a decline in MP, MI, and PsyCap. Exploring the relationships between IA, MI, PsyCap, mental health, and MP can provide a better understanding of how these variables influence and interact, ultimately contributing to the development of strategies and interventions to promote ethical behavior in the digital age [46]. Exploring unexplored areas can provide new insights, expand our understanding of phenomena, and generate valuable knowledge. For example, a systematic review was conducted outside of Europe and discovered a lack of studies originating from Africa and South America [47]. Most of the research in this area has been conducted in Western cultures, particularly in North America and Asia. Cultural differences in values, norms, and technology use patterns may influence the relationship between IA, MP, and other psychological variables. Therefore, there is a great need for more studies that explore the impact of IA and related factors in diverse cultural contexts [12,46,47,48,49].

Second, although studies have identified associations between IA, MI, PsyCap, and MP relationships [1,11,12,18,35,37], there is a need for research that investigates the underlying mechanisms and mediators of these relationships. While some studies have explored the negative impact of IA on MP, there is a need for more research studying other mediating impacts on this relationship between IA and MP. Understanding the factors contributing to IA problems can inform the development of evidence-based interventions promoting healthy online behaviors, MI, and MP [8,12,21,34,50,51,52]. While existing research has shed light on the relationships between these variables, several research gaps remain. Further investigation is needed to explore the underlying mechanisms, mediating factors, and cross-cultural differences to develop effective strategies for prevention and intervention. By addressing these research gaps, we can better understand the impact of IA on MP and promote ethical behavior in the digital age.

Building on the previous research, this study aims to explore the predictive relationship between internet addiction (IA) and moral psychology (MP), while also investigating the potential mediating roles of psychological capital (PsyCap) and moral identity (MI). Drawing on theoretical frameworks such as the cognitive–behavioral model of IA [53], the social cognitive theory of moral development [54], the broaden-and-build theory of positive emotions [55], and positive psychology theory [56], the researchers seek to elucidate the complex interplay among these key constructs.

By addressing these important research gaps, this study aims to enhance our understanding of the intricate connections between IA, MI, PsyCap, and moral psychology more broadly. This knowledge can then inform the development of targeted interventions, policies, and educational programs to promote healthier and more balanced digital technology use patterns among individuals and within society [57]. Ultimately, this research represents a timely and critical step in unraveling the nuanced relationships between problematic internet use, moral capacities, psychological resources, and overall moral functioning. The findings have the potential to guide evidence-based strategies to support flourishing in the digital age.

Overall, to the best of our knowledge, except for the role of these comprehensive mediation models, the role of IA and social media overuse on MP mediated through positive PsyCap and MI has not been examined. Therefore, to fill this gap, it is essential to conduct empirical research to determine the nature and strength of these relationships.

### 2.5. Study Rationale

**Figure 1:**
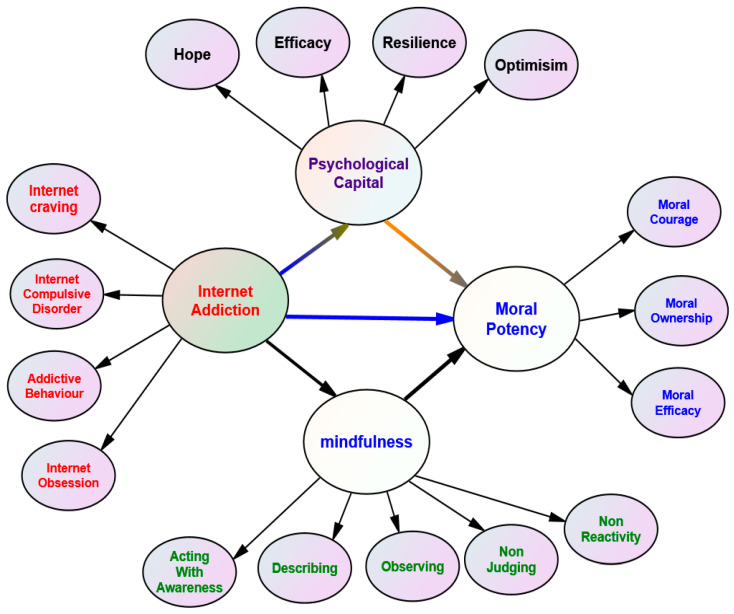
A theoretical model of IA’s impact on MP, mediated by MI and PsyCap (Psychological Capital). *Note.* The figure illustrates the proposed relationships between the variables in the theoretical model.

This study aims to explore the relationship between IA, MI, PsyCap, and MP among students. It hypothesizes that IA negatively impacts students’ moral development, while MI and PsyCap play a mediating role in this relationship. This study further proposes that higher levels of MI, PsyCap, and MP can reduce IA and enhance moral development among students. Based on the most recent scientific literature and the constructed theoretical framework depicted in Figure 1, this study addressed the following research questions.

**RQ1:** What is the association between MI, PsyCap, and MP?
**RQ2:** What are the differences among the university types regarding students’ IA, MI, PsyCap, and MP?
**RQ3:** What are the impacts (direct and mediating) of students’ IA, MI, and PsyCap on their MP?

## 3. Materials and Methods

### 3.1. Research Design

The current study employed a quantitative research design with an associational design, which was well suited to achieving the stated objectives.

### 3.2. Sample and Sampling

The target population for this study comprised undergraduate students from public universities in the Amhara Regional State. Specifically, this study was conducted at three randomly selected public universities: Gondar University, Wollo University, and Woldia University. To recruit participants, a simple random sampling technique was employed. This study was conducted at three randomly selected public universities in the Amhara Regional State: Gondar University, Wollo University, and Woldia University. This research began with a randomly selected sample of 889 university students who were invited to take part in the surveys. However, due to various issues like incomplete information, data entry errors, or participant carelessness, 21 of these individuals had to be excluded from the final analysis. Despite these necessary exclusions, this study ultimately maintained a highly robust effective response rate of 97.6%. This means the final sample size consisted of 868 participants out of the original 889 students.

The final sample consisted of 868 undergraduate students, with 526 male students (60.6%) and 342 female students (39.4%). The participants had a mean age of 22.31, with a standard deviation of 4.03. Among the participants, 284 (32.7%), 246 (28.3%), and 338 (38.9%) students belonged to Gondar University (Research), Wollo University (Applied), and Woldia University (Comprehensive), respectively.

To comprehend the differences among the students’ IA, MI, PsyCap, and MP beyond the variations in university types, the students were further categorized by their year of study or batch, and their differences were studied. Specifically, 238 students were classified as Freshman (1st year), 267 students as Sophomore (2nd year), and 363 students as Senior (>2nd year). These selections were made using a simple random sampling technique. Undergraduate students self-reported their gender, batch (year of study), age, and university.

### 3.3. Instruments

This study investigated the relationships among IA, MI, PsyCap, and MP of university students as shown in the theoretical model (Figure 1). Therefore, four main instruments were applied in this study, each selected for their relevance to the constructs being measured and their importance to the research questions.

#### 3.3.1. Internet Addiction Scale (IAS)

Sondhi and Joshi [16] developed the 17-item IAS to assess an individual’s excessive and compulsive internet use that interferes with their daily functioning. The scale utilizes a 7-point response format ranging from “Very Strongly Disagree” (1) to “Very Strongly Agree” (7). The IAS encompasses four core dimensions that demonstrate sound psychometric properties: (1) Internet Craving (IC)—5 items, α = 0.771, CR = 0.836; (2) Internet Compulsive Disorder (ICD)—4 items, α = 0.776, CR = 0.857; (3) Addictive Behavior (AB)—4 items, α = 0.741, CR = 0.802; and (4) Internet Obsession (IO)—4 items, α = 0.663, CR = 0.862. The overall IAS scale exhibits strong internal consistency, with a Cronbach’s alpha of 0.878. Furthermore, the IAS measure also had strong reliabilities and construct validity in Ethiopian settings used in the current study [12]. This multifaceted instrument provides a comprehensive assessment of problematic internet use that extends beyond just time spent online. The robust psychometric qualities of the IAS underscore its reliability and validity for researchers and clinicians evaluating maladaptive internet engagement. Therefore, the good psychometric properties of the scale and multifaceted nature make it a reliable tool for measuring IA, which is central to the research question regarding the impact of IA on students’ cognitive and psychological states.

#### 3.3.2. Psychological Capital Questionnaire (PCQ-24)

The PCQ-24 is a 6-point Likert scale developed by Luthans et al. [58] to assess respondents’ positive psychological resources. It consists of four subscales: hope, self-efficacy, optimism, and resilience. Each subscale has 6 items. For the current study, the researchers used the Ethiopian version of the PCQ-24, which had been adapted to ensure psychometric suitability in the Ethiopian context. The internal consistency reliability (Cronbach’s alpha) of the subscales was as follows: (a) hope: α = 0.80, 0.72, 0.75, 0.76; (2) self-efficacy: α = 0.84, 0.75, 0.85, 0.75; (3) optimism: α = 0.76, 0.74, 0.69, 0.79; and (4) resilience: α = 0.71, 0.66, 0.71, 0.72. The overall reliability of the PsyCap scale was α = 0.89, 0.88, 0.89, 0.89. The PCQ-24 was chosen because PsyCap is a critical construct in the theoretical model, hypothesized to mediate the relationship between IA and students’ psychological well-being.

#### 3.3.3. Five Facet Mindfulness Questionnaire Short Form (FFMQ-SF)

The FFMQ-SF developed by Baer et al. [25] and later modified by Hou et al. [59] was used in this study and aimed to assess MI levels. The FFMQ-SF has five subscales, each with four items—acting with awareness (α = 0.77), describing (α = 0.84), observing (α = 0.81), non-judging (α = 0.69), and non-reactivity (α = 0.70)—as well as the total scale (α = 0.85). Respondents’ responses are given on a 5-point Likert scale ranging from 1 (very rarely true) to 5 (almost always true). MI is a crucial variable in this study, hypothesized to mitigate the negative effects of IA on students’ PsyCap and MP.

#### 3.3.4. MP Questionnaire (MPQ)

The MPQ is a theoretically grounded scale that covers the full range of human moral development and concerns. The authors developed the MPQ to assess an individual’s capacity to act morally and make ethical decisions using a 12-item scale [8] The questionnaire consists of three dimensions: moral courage (four items; α = 0.79), moral ownership (four items; α = 0.85), and moral efficacy (four items; α = 0.92). The overall MP is measured with an alpha coefficient of 0.85. Respondents are asked to indicate their agreement with statements on a 5-point Likert scale, ranging from 1 (Strongly Disagree) to 5 (Strongly Agree). The MPQ was chosen because it directly measures the moral aspects hypothesized to be influenced by IA and related to students’ psychological and cognitive development.

### 3.4. Statistical Data Analysis

For data analysis, this study employed various statistical software packages, including SPSS version 29 and SmartPLS 4.1.0.3. The researchers assessed the presence of multicollinearity issues in the data by utilizing measures such as the Variance Inflation Factor (VIF) and tolerance [47,60,61]. The maximum likelihood estimation was employed to determine the measurement and structural relationships within the proposed model. The goodness-of-fit of the models was evaluated using several indices, including the normed chi-square (χ^2^/df), Tucker–Lewis Index (TLI), Comparative Fit Index (CFI), Standardized Root Mean Residual (SRMR), and Root-Mean-Squared Error of Approximation (RMSEA). Generally, an excellent and satisfactory model fit is indicated by χ^2^/df values below 3 or 5, RMSEA and SRMR values below 0.08 and 0.01, and TLI and CFI values above 0.95 and 0.90, respectively [60,61,62]. These indices provide information on how well the proposed models align with the observed data, with lower χ^2^/df, RMSEA, and SRMR values and higher TLI and CFI values indicating a better fit [63,64]. To examine indirect effects, the researchers performed bootstrap analysis with 5000 resamples to calculate 95% bias-corrected and accelerated confidence intervals.

### 3.5. Procedures of the Studies

This study followed the ethical guidelines set by the American Psychological Association, ensuring voluntary participation and adherence to the Helsinki Declaration, including the 21 CFR 50 and 21 CFR 56 regulations, for the protection of human subjects and institutional review boards. The researchers placed great emphasis on maintaining confidentiality and anonymity of the participants and their data. This study identified multiple internal and external factors influencing moral potency (MP), and thus, internet addiction (IA) was targeted to modify two mediators, psychological capital (PsyCap) and mindfulness (MI), in order to enhance MP. A study conducted by Mortimer et al. [65] highlighted five mindfulness strategies, including acting with awareness, describing, observing, non-judging, and non-reactivity, as the main focus for improving students’ MI skills. PsyCap encompasses four key components: hope, efficacy, resilience, and optimism. In this study, the total MI and PsyCap targeting all students’ MP mediated the relationship between IA and MP. The multiple mediator models in psychological studies serve as the theoretical basis for various intervention and association studies [8,15,51,65].

## 4. Results

This study addressed three research questions focusing on the associations among the variables of IA, MI, PsyCap, and MP; group differences of students; and the impacts of students’ IA, MI, and PsyCap on their MP. Firstly, a preliminary analysis using descriptive statistics was conducted to inquire about the frequency distribution of the assessments. Secondly, multicollinearity analysis was performed to detect potential multicollinearity effects among the variables for subsequent correlational analyses. Thirdly, correlational analyses were carried out to explore the associations among the IA, MI, PsyCap, and MP. Fourth, students’ group differences were investigated across these variables. Lastly, the mediation analyses were conducted to determine the indirect effects of MI and PsyCap on the relationship between IA and MP. 

### 4.1. Results of Preliminary Analysis

Table 1 provides the descriptive statistics, including the mean and standard deviation, for the different variables in this study. The absolute values of skewness and kurtosis for the variables (IA, PsyCap, MI, and MP) are within the acceptable range for a normal distribution (skewness ≤ 2, kurtosis ≤ 4) recommended by Kim [66]. In this study, the skewness values for IA, PsyCap, MI, and MP were found to be −1.04, −0.46, −0.85, and −0.96, respectively. The kurtosis values for these constructs were 1.49, 0.29, 1.10, and 0.95, respectively.

### 4.2. Multi-Collinearity

After confirming the normality of the data through skewness and kurtosis, the researchers assessed the absence of multicollinearity among the predictor variables. Researchers [60] suggest that if the tolerance values of predictor variables in a model are close to each other, it indicates the absence of multicollinearity. The Variance Inflation Factor (VIF) is another statistic used to assess multicollinearity in a dataset. Ideally, VIF values should range from 0 to 5, with lower values being more desirable. If the VIF for a variable exceeds 5, it indicates that the variable is a linear combination of other predictor variables in the model [60]. In the current study, the researchers found that the VIF values for all independent variables were below 5. Additionally, the tolerance limits for each independent variable were greater than or equal to 0.01. This suggests that multicollinearity was not a significant issue in the dataset used for this study. Based on these measures, the researchers concluded that multicollinearity was not an issue (see Table 2).

In addition, to assess the potential impact of common method bias, the researchers conducted Harman’s single-factor test. This analysis examines whether a single factor accounts for the majority of the variance in the data, which would indicate the presence of common method bias. The results of Harman’s single-factor test showed that all the constructs had a common method bias rate of 28.36%. This value was below the recommended threshold, suggesting that common method bias was not a significant issue in this study [12,47]. Based on these findings, the researchers concluded that the study results were not substantially impacted by bias stemming from common method variance. This provided confidence that the observed relationships between the variables were not unduly influenced by shared method variance.

### 4.3. Correlational Analysis

After confirming the normality of the data distribution and the absence of multi-linearity, the researchers examined the relationships between sociodemographic factors and the study variables using bivariate correlation analysis. Gender, age, and batch (year of study) were found to have non-significant relationships with the study variables and were therefore excluded from further analysis. Pearson’s correlations were conducted to determine the relationships between university type (a sociodemographic factor) and the study variables (see Table 3).

The results revealed significant correlations between university type and IA (*r* = 0.08, *p* < 0.05), MI (*r* = 0.20, *p* < 0.01), PsyCap (*r* = 0.15, *p* < 0.01), and MP (*r* = 0.34, *p* < 0.01), indicating that different types of universities were associated with differences in these variables. Specifically, IA displayed a significant negative correlation with MI (*r* = −0.14, *p* < 0.01), PsyCap (*r* = −0.28, *p* < 0.01), and MP (*r* = −0.29, *p* < 0.01). MI showed a positive and significant correlation with PsyCap (*r* = 0.39, *p* < 0.01) and MP (*r* = 0.45, *p* < 0.01), while PsyCap exhibited a positive and significant relationship with MP (*r* = 0.50, *p* < 0.01).

### 4.4. Group Differences

To further analyze the differences between university types, the researchers conducted one-way ANOVA tests with a multi-group comparison (Tukey’s Multi-Comparison) test. This study found statistically significant differences across university types, as demonstrated by one-way ANOVA analyses for Internet Addiction (IA), mindfulness (MI), psychological capital (PsyCap), and Moral Potency (MP). Specifically, the ANOVA tests revealed significant group differences for IA (F(867) = 3.09, *p* = 0.046), MI (F(867) = 18.18, *p* = 0.001), PsyCap (F(867) = 14.32, *p* = 0.001), and MP (F(867) = 10.67, *p* = 0.001). While the initial F-test (reported in Appendix A) indicated significant differences among participants across age categories, this study was unable to pinpoint the exact source of these differences. To further explore the significant ANOVA findings, the researchers employed Tukey’s pairwise comparison test (results provided in Appendix B).

The Tukey post hoc analysis showed a significant mean difference between research universities and comprehensive universities for three of the measured constructs: IA (mean diff = −2.69, *p* < 0.05), PsyCap (mean diff = −8.65, *p* < 0.05), and MP (mean diff = −4.27, *p* < 0.05). These findings suggest that the type of university attended (research-focused vs. comprehensive) was associated with meaningful differences in students’ levels of internet addiction, psychological capital, and moral potency.

In addition, a mean score difference was found between research universities with an applied university and a comprehensive university (mean diff = −5.81 *, −8.19 *) in the MI construct, respectively. In addition, differences were found between an applied university and comprehensive university in IA (mean diff = −2.39 *), PsyCap (mean diff = −10.22 *), and MP (mean diff = −4.34 *). However, research universities and applied universities in IA, PsyCap, and MP and applied universities and comprehensive universities in IA had no significant mean differences.

Moreover, a research university had a significantly lower score in IA and a higher score in MI, PsyCap, and MP (M = 62.88, 79.93, 94.54, 51.35; SD = 15.44, 14.77, 17.78, 10.29) compared to the applied university (M = 64.80, 77.54, 84.32, 47.01; SD = 14.79, 17.83, 30.57, 16.41) and comprehensive university (M = 62.88, 71.74, 85.89, 47.07; SD = 15.44, 19.07, 28.02, 13.71), respectively. In addition, students in an applied university had a higher level of MI, PsyCap, and MP and a lower level of IA than students in a comprehensive university.

### 4.5. Mediation Analysis

**Figure 2:**
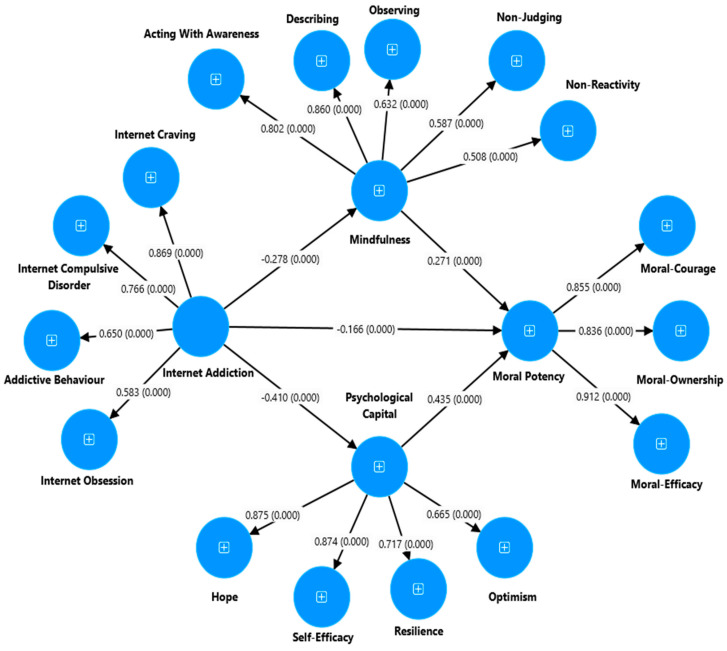
Full mediation model: the mediating role of MI and PsyCap between IA and MP (result).

This study examined IA and MP through MI and PsyCap (Figure 2). The variance explained (R^2^) proportion was used to examine the accuracy of the prediction power of independent variables on dependent variables of the structured model obtained from the data. As a result, the model accounts for 40.1 percent of the variance in MP, 16.8 percent of the variance in PsyCap of university students, and 7.7 percent of the variance in MI of university students.

The results from Table 4 also show that the standardized direct effect path from IA to MI, PsyCap, and MP was negative and statistically significant (β = −0.28, [BC 95% bootstrap CI: −0.35 to −0.20], *p* = 0.002), (β = −0.41 [95% bootstrap CI: −0.49 to −0.33], *p* = 0.002), and (β = −0.17 [95% bootstrap CI: −0.23 to −0.10], *p* = 0.002), respectively. Furthermore, the results of this study support the stated hypothesis 5 in which the direct effect of MI and PsyCap in students’ MP was significant and positive (β = 0.27 [95% bootstrap CI: 0.19 to 0.37], *p* = 0.002) and (β = 0.43 [95% bootstrap CI: 0.34 to −0.51], *p* = 0.002), respectively.

**Figure 3:**
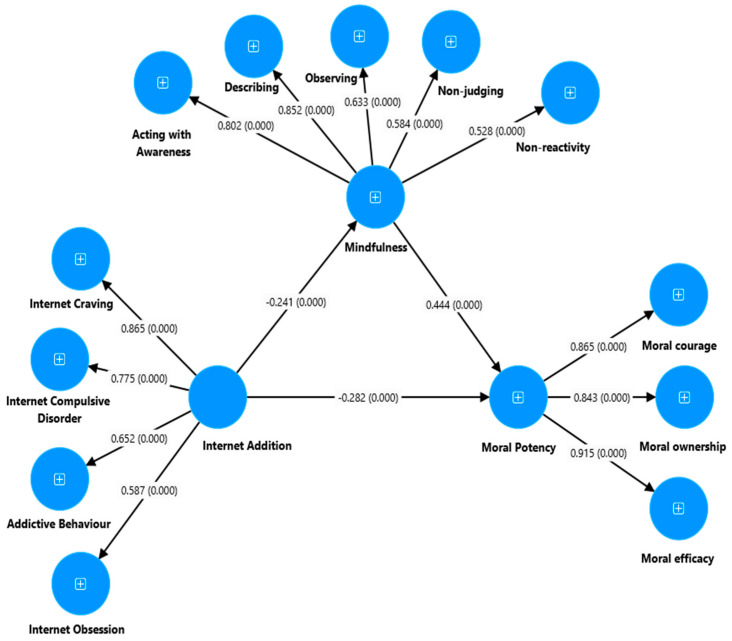
Partial mediation model: the mediating role of MI between IA and MP (result). *Note.* A partial mediation model of MI in the relationship between IA and MP.

The next step involved examining the partial mediation by considering students’ MP as the dependent variable, IA as the predictor variable, and MI as the mediator variable (Figure 3). This study found that IA had a significant negative direct effect on students’ MI (β = −0.24, 95% bootstrap CI: −0.31 to −0.16, *p* = 0.003) and MP (β = −0.28, 95% bootstrap CI: −0.35 to −0.22, *p* = 0.002). Additionally, the direct effect of MI on students’ MP was positive and significant (β = 0.44, 95% bootstrap CI: 0.36 to 0.53, *p* = 0.003). The partial mediation model, examining the indirect effect of IA on students’ MP through MI, was also negative and significant (β = −0.11, 95% bootstrap CI: −0.15 to −0.07, *p* = 0.002). The mediation model through MI demonstrated an acceptable structural model fit based on various fit indices: χ^2^(1112) = 5310, *p* < 0.001, χ^2^/df = 4.77, TLI = 0.90, CFI = 0.91, SRMR = 0.04, and RMSEA = 0.07 (0.06 to 0.07). These findings indicate that the model’s structural validity was acceptable, consistent with the cut-off points suggested by Hu and Bentler [64]. In summary, the results demonstrate a significant direct impact of IA on both MI and MP among students. Additionally, MI has a positive and significant direct effect on students’ MP. Moreover, this study confirms the presence of partial mediation, indicating that MI partially mediates the relationship between IA and students’ MP. The overall model fit was deemed acceptable based on various fit indices, supporting the validity of the model.

The indirect effect of IA on university students’ MP mediated through MI and PsyCap was significant (β = −0.25, [95% bootstrap CI: −0.31, −0.20], *p* = 0.002) (Table 5). The structural model of this mediation indicates a good model fit (see χ^2^ (1782) = 44,483, *p* < 0.001, TLI = 0.92, CFI = 0.93, SRMR = 0.02, and RMSEA = 0.04 (0.04 to 0.05). The measurement model goodness-of-fit also had an acceptable fit: χ^2^ (1799) = 4461, *p* = 0.001, TLI = 0.92, CFI = 0.93, SRMR = 0.02, and RMSEA = 0.04 (0.04 to 0.05). This result implies that this research model has an acceptable structural and measurement validity, supported by the cut-off points prescribed by Hu and Bentler [62]. 

**Figure 4:**
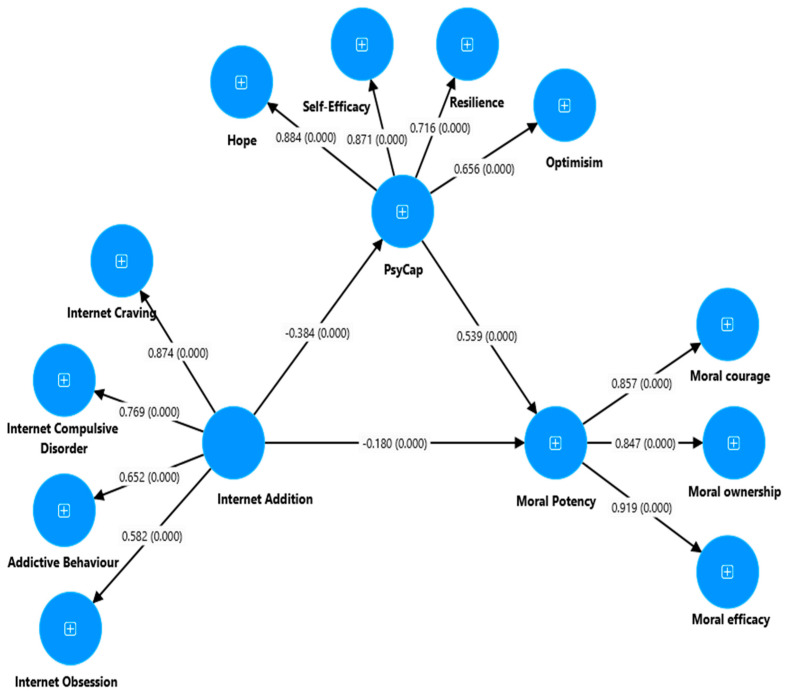
Partial mediation model: the mediating role of PsyCap between IA and MP (result). *Note.* Figure 4 indicates the partial mediation output mode on the mediating role of PsyCap between IA and students’ MP.

Furthermore, IA had a negative and significant indirect effect on students’ MP through PsyCap (β = −0.21, 95% bootstrap CI: −0.26 to −0.15, *p* = 0.002) (Figure 4). This study also found a significant negative direct effect of IA on students’ PsyCap (β = −0.38, 95% bootstrap CI: −0.46 to −0.30, *p* = 0.002), as well as a negative direct effect of IA on MP (β = −0.18, 95% bootstrap CI: −0.25 to −0.11, *p* = 0.002). Additionally, the direct effect of PsyCap on students’ MP was positive and significant (β = 0.54, 95% bootstrap CI: 0.47 to 0.61, *p* = 0.001). The model demonstrated an acceptable goodness-of-fit with index values for the structural model: χ^2^ (1311) = 4971.99, *p* < 0.001, χ^2^/df = 3.79, TLI = 0.92, CFI = 0.92, SRMR = 0.03, and RMSEA = 0.06 (0.05 to 0.06). A GFI, RFI, TLI, and CFI value of 0.90 indicates an acceptable fit for the model, while values of 0.95 and above indicate an excellent fit, as recommended by Hu and Bentler (1999).

## 5. Discussion

This study investigated the relationships between sociodemographic factors, IA, MI, PsyCap, and MP among Ethiopian university students. Three research questions were addressed in this study.

The first research question focused on the relationship between IA and MI, PsyCap, and MP. The results revealed a significant negative correlation between IA and MI, PsyCap, and MP among university students. These findings are consistent with previous studies that have reported similar negative relationships [11,21,34,35,36], highlighting the detrimental impact of IA on these psychological factors. However, it is worth noting that when used in the correct way, IA can have positive impacts on building virtual interactions among people [67] and students’ academic achievement [68]. Moreover, this study provides new evidence specific to the Ethiopian context, which has been underrepresented in the literature. The negative relationship between IA and these psychological constructs in this unique cultural and educational setting expands the generalizability of previous findings.

The second research question explored group differences based on university classification. One-way ANOVA with Tukey’s HSD multiple group comparisons revealed significant differences in IA, MI, PsyCap, and MP levels across university classifications. The results suggested that as the university classification improved, MI, psychological resources, and MP increased, while IA decreased. These findings align with previous research, indicating that sociodemographic factors, such as university classification, significantly affect the well-being of university students [8,15,69]. However, it is worth noting that the literature presents inconsistent findings, emphasizing the need for further research to gain a more comprehensive understanding of these relationships and differences. Contrary to some studies [41,42] that suggest a more uniform impact of IA across different educational settings or genders, our results indicate that the classification of the university can significantly influence these variables. This divergence indicates the need for further research to understand how institutional factors contribute to these differences.

The third research question concerns the impacts of students’ IA (both direct and mediating effects) together with PsyCap and MI on their MP. This study also examined whether IA negatively affects MI, PsyCap, and MP. Hence, this study found that IA is a negative predictor of MI, PsyCap, and MP. Here, the results are consistent with other studies reported in the scientific literature [11,21,34,35,36,70]. This study also examined whether MI and PsyCap are positive and significant predictors of MP. The findings confirmed that MI is a significant positive predictor of MP. While some studies (e.g., [11,30]), support the idea that mindfulness is positively related to moral variables, others suggest that the nature and mechanisms of this relationship are not fully understood and require further investigation [29,71].

This study also investigated the impact of internet addiction (IA) on students’ moral potency (MP) by considering the mediating effects of mindfulness (MI) and psychological capital (PsyCap). The results revealed that both MI and PsyCap fully mediated the relationship between IA and MP. These findings align with existing research in the scientific literature, which consistently highlight the negative role of IA [11,12,21,34,35,70]. Additionally, previous studies conducted by other researchers [12,33,34] have established a connection between addiction to social networking sites (SNSs), higher levels of IA, increased moral disengagement, and poorer mental health. These findings hold substantial implications for interventions and preventive measures aimed at addressing IA and fostering moral well-being among university students.

The partial mediation model also confirmed whether MI mediates the relationship between IA and MP. This implies that a higher MI leads to a lower IA and a better MP in university students. While the existing literature supports the role of MI as a mediator, our study contributes new insights by confirming that a higher MI leads to a lower IA and better MP among Ethiopian University students. This protective role of MI aligns with findings that indicate that MI helps mediate adverse life experiences and promotes good moral judgment [71]. Finally, this study determined whether PsyCap plays a mediating role in the relationship between IA and students’ MP. We found that PsyCap has played a partial mediator role in the relationship between IA and MP, or IA had an indirect, negative, and significant effect on MP through PsyCap. Previous studies have discussed the protective role of positive psychological resources [27,70]. For example, psychological resources improve the MP of employees [7,27,72,73]. Our study adds a new dimension by specifically linking these findings to the context of Ethiopian University students. This highlights the importance of fostering psychological capital to mitigate the negative effects of IA.

The findings of this study contribute valuable insights into the impact of IA on MP in university students. This study emphasizes the importance of addressing IA and promoting MI and PsyCap as key factors in enhancing MP among this population. These results highlight the need for positive interventions that focus on improving moral well-being, promoting healthy internet usage, and fostering positive psychological resources such as hope, efficacy, resilience, and optimism among university students.

To sum up, this study provides valuable insights into fulfilling the research gaps, such as the scarcity of this associational research (based on IA, MI, PsyCap, and MP) in Ethiopian and European contexts, and the need for investigating the underlying mechanisms and mediators (MI and PsyCap) of the relationships between IA and MP. This study confirms several established relationships while also providing new contributions to the literature by highlighting the unique aspects of these relationships in the Ethiopian context and the significant mediating roles of MI and PsyCap. The findings shed light on the importance of addressing IA and promoting positive resources such as MI and PsyCap to enhance MP, by explicitly addressing the effect of IA on MP and highlighting the need for targeted interventions that consider cultural and institutional contexts. The study implications extend to the development of interventions aimed at improving students’ moral well-being and promoting healthy internet usage. Therefore, the further exploration of these relationships and their impacts is of great theoretical and practical value.

## 6. Conclusions

The results revealed significant negative relationships between IA and MI, PsyCap, and MP. Moreover, some significant differences were found among the students’ MP across different university types. This study also proved that IA had a significant and negative direct effect on MI, PsyCap, and MP. Moreover, MI and PsyCap had a positive direct impact on MP and fully mediated the relationship between IA and MP. These findings indicate that higher levels of IA among university students are associated with lower levels of MI, PsyCap, and MP. The finding that MI and PsyCap fully mediate the relationship between IA and MP suggests that the detrimental effect of IA on MP primarily stems from its impact on positive MI and PsyCap resources. In other words, when university students experience higher levels of IA, it leads to a lower MI and PsyCap, which, in turn, contributes to poorer moral well-being outcomes. These results underscore the importance of addressing IA in university students, as it not only directly affects MI, PsyCap, and MP but also indirectly influences MP through its impact on MI and PsyCap. Therefore, it is crucial to implement interventions and preventive measures that target both IA and the promotion of MI and PsyCap resources among university students. By reducing IA and fostering MI and PsyCap, we can enhance the MP of university students and mitigate the negative effects of IA on MP.

Overall, the findings emphasize the need for a comprehensive approach to addressing the interplay between IA, MI, PsyCap, and MP among university students. By targeting these factors simultaneously, we can develop effective strategies to enhance MP outcomes and promote overall moral well-being in this population.

### 6.1. Limitations and Future Research Recommendations

This study will have a potential role by applying a positive psychology model to minimize the adverse effects of IA and boost university students’ MP. However, this study has the following limitations.

- *Cross-sectional design.* This study employed an associational design, which limits the ability to infer causality among the variables over time. A longitudinal study would help establish the directionality of the relationships over time.
- *Self-report measures*. All the data were collected through self-report measures, which may be subject to social desirability bias and common method variance, even though common method bias was checked in this study. Future studies could incorporate multi-source or multi-method approaches to data collection.
- *Generalizability.* This study was conducted with university students in Ethiopia, which may limit the generalizability of the findings to other populations or cultural contexts. Replicating this study in different settings or with diverse samples would enhance the external validity.
- *Measurement of IA.* This study used a general measure of IA, but more specific measures focusing on different types of problematic internet use (e.g., social media addiction, gaming addiction) could provide more nuanced insights.

### 6.2. Future Research Recommendations

This study recommended the following for future research.

- Longitudinal studies. Conducting longitudinal studies would allow researchers to investigate the dynamic and reciprocal relationships between the variables over time, providing a more robust understanding of the underlying processes.
- Mixed-methods approach. Incorporating qualitative methods, such as interviews or focus groups, could provide in-depth insights into the lived experiences and perspectives of university students regarding IA, MI, PsyCap, and MP.
- Moderating and mediating factors. Examining potential moderating or mediating variables (e.g., social support, academic engagement, family factors) could help elucidate the complex mechanisms underlying the relationships between the studied constructs.
- Intervention studies. Developing and evaluating the effectiveness of interventions aimed at reducing IA and promoting MI, PsyCap, and MP among university students would have significant practical implications.
- Cross-cultural comparisons. Conducting comparative studies across different cultural contexts would enhance the understanding of how sociocultural factors may influence the relationships between the variables.
- Objective measures. Incorporating objective measures of internet usage, cognitive processes, and moral behavior could complement the self-report data and provide a more comprehensive understanding of the phenomena.

## Figures and Tables

**Table 1:** Descriptive statistics, kurtosis, and skewness.

Variables	Min	Max	Mean	Std. Dev.	Skewness	Kurtosis
Internet craving	5.00	28.00	19.97	4.86	−1.09	0.70
Internet compulsive disorder	4.00	22.00	14.73	4.18	−0.74	−0.18
Addictive behavior	4.00	20.00	15.40	3.95	−0.93	0.22
Internet obsession	4.00	20.00	14.36	4.76	−0.61	−0.74
Internet addiction	17.00	90.00	64.48	13.69	−1.04	1.49
Hope	6.00	36.00	23.73	7.61	−0.84	0.00
Efficacy	6.00	36.00	21.64	8.15	−0.26	−0.75
Resilience	5.00	36.00	22.10	7.62	−0.32	−0.61
Optimism	6.00	36.00	21.33	7.74	−0.35	−0.74
Psychological capital	23.00	144.00	88.81	25.78	−0.46	0.29
Acting with awareness	4.00	24.00	16.44	4.79	−0.79	0.289
Describing	4.00	24.00	16.57	4.97	−0.85	0.20
Observing	4.00	24.00	14.32	4.89	−0.33	−0.62
Non-judging	4.00	24.00	13.04	4.67	−0.15	−0.69
Non-reactivity	4.00	25.00	16.21	4.78	−0.52	−0.10
Mindfulness	20.00	118.00	76.58	17.48	−0.85	1.10
Moral ownership	4.00	25.00	16.59	5.00	−0.85	0.27
Moral courage	4.00	25.00	15.53	4.94	−0.51	−0.24
Moral efficacy	4.00	25.00	16.60	5.02	−0.85	0.18
Moral potency	12.00	74.00	48.72	13.53	−0.96	0.95

**Table 2:** Tolerance and VIF of multi-collinearity statistics (Dependent variable: Moral potency).

Model	Unstandardized Coefficients	Standardized Coefficients	t	Sig.	Collinearity Statistics	
	Beta	Beta			Tolerance	VIF
Internet addiction	−0.14	−0.14	−5.05	<0.001	0.92	1.09
Psychological capital	0.18	0.35	11.24	<0.001	0.79	1.26
Mindfulness	0.23	0.30	9.96	<0.001	0.85	1.18

**Table 3:** Pearson correlations (r) among sociodemographic factors, predictor variables, and criterion variables (N = 868).

Variables	Moral Courage	Moral Ownership	Moral Efficacy	Moral Potency
Sex	−0.04.	−0.08 *	−0.01	−0.05
Age	−0.04	0.01	−0.09 *	−0.04
University	0.29 **	0.33 **	0.29 **	0.34 **
Batch	0.03	0.00	0.01	0.02
Internet craving	−0.21 **	−0.20 **	−0.24 **	−0.24 **
Internet compulsive disorder	−0.22 **	−0.20 **	−0.20 **	−0.23 **
Addictive behavior	−0.14 **	−0.09 **	−0.17 **	−0.15 **
Internet obsession	−0.21 **	−0.27 **	−0.19 **	−0.25 **
Internet addiction	−0.26 **	−0.25 **	−0.26 **	−0.29 **
Hope	0.47 **	0.44 **	0.51 **	0.52 **
Efficacy	0.42 **	0.41 **	0.44 **	0.47 **
Resilience	0.38 **	0.39 **	0.38 **	0.42 **
Optimism	0.24 **	0.20 **	0.24 **	0.25 **
Psychological capital	0.46 **	0.43 **	0.47 **	0.50 **
Acting with awareness	0.31 **	0.18 **	0.29 **	0.29 **
Describing	0.28 **	0.25 **	0.29 **	0.30 **
Observing	0.13 **	0.13 **	0.13 **	0.15 **
Non-judging	0.25 **	0.15 **	0.21 **	0.22 **
Non-reactivity	0.74 **	0.53 **	0.57 **	0.68 **
Mindfulness	0.47 **	0.34 **	0.41 **	0.45 **

*Note.* * (*p* < 0.05), ** (*p* < 0.01).

**Table 4:** A standardized direct effect of IA, MI, and PsyCap on MP.

Dependent Variables	Path	Independent Variables	Standardized Direct Effect	Bootstrap 95% CI		
				Lower Bound (LBC)	Upper Bound (UBC)	*p*-Value
MI (R^2^ = 0.077)	←	IA	−0.28	−0.35	−0.20	0.002
PsyCap (R^2^ = 0.168)	←	IA	−0.41	−0.49	−0.31	0.002
MP (R^2^ = 0.401)	←	IA	−0.17	−0.23	−0.10	0.002
MP	←	MI	0.27	0.19	0.37	0.002
MP	←	PsyCap	0.43	0.34	0.51	0.002
*Total Direct Effect of IA on MP through MI and PsyCap*						
MI	←	IA	−0.28	−0.35	−0.20	0.002
PsyCap	←	IA	−0.41	−0.49	−0.33	0.002
MP	←	IA	−0.42	−0.48	−0.35	0.002
MP	←	MI	0.27	0.19	0.37	0.002
MP	←	PsyCap	0.43	0.34	0.51	0.002
*Partial Mediation: Direct Effect of IA on MP through MI*						
MI	←	IA	−0.24	−0.31	−0.16	0.003
MP	←	IA	−0.28	−0.35	−0.22	0.001
MP	←	MI	0.44	0.036	0.53	0.003
*Partial mediation: Direct Effect of IA on MP through PsyCap*						
PsyCap	←	IA	−0.38	−0.46	−0.30	0.002
MP	←	IA	−0.18	−0.25	−0.11	0.002
MP	←	PsyCap	0.54	0.47	0.61	0.001

*Note.* CI = confidence interval, LBC = lower bound, UBC = upper bound, IA (internet addiction), PsyCap (psychological capital), MI (mindfulness), MP (moral potency).

**Table 5:** Bootstrapping standardized indirect effect using 95% bias-corrected confidence interval predicting students’ MP (N = 868).

Path Model		Bootstrap 95% CI		
	Beta	LBC	UBC	*p*-Value
IA → MI and PsyCap → MP	−0.25	−0.31	−0.20	0.001
IA → MI → MP	−0.11	−0.15	−0.07	0.002
IA → PsyCap → MP	−0.21	−0.26	−0.15	0.002

*Note.* CI = confidence interval, LBC = lower bound, UBC = upper bound, IA (internet addiction), PsyCap (psychological capital), MI (mindfulness), MP (moral potency).

## Data Availability

The corresponding authors hold the datasets generated and analyzed during this study and are willing to share them upon request.

## Appendix A

Summary of ANOVA results of the three university classifications

**Table:**

ANOVA										
Variable University Types		N	Mean	Std. Deviation		Sum of Squares	df	Mean Square	F	Sig.
IA	Research University	284	62.88	15.44	Between Groups	1154.70	2	577.35	3.09	0.046
	Comprehensive University	246	64.80	14.79	Within Groups	161,547.84	865	186.76		
	General University	338	65.57	10.95	Total	162,702.54	867			
MI	Research University	284	79.93	14.77	Between Groups	10,686.67	2	5343.33	18.18	<0.001
	Comprehensive University	246	77.54	17.83	Within Groups	254,248.84	865	293.93		
	General University	338	71.74	19.07	Total	264,935.51	867			
PsyCap	Research University	284	94.53	17.78	Between Groups	18,468.51	2	9234.26	14.32	<0.001
	Comprehensive University	246	84.32	30.57	Within Groups	557,663.24	865	644.69		
	General University	338	85.89	28.02	Total	576,131.76	867			
MP	Research University	284	51.35	10.29	Between Groups	3821.85	2	1910.93	10.67	<0.001
	Comprehensive University	246	47.01	16.42	Within Groups	154,937.33	865	179.12		
	General University	338	47.07	13.72	Total	158,759.19	867			

## Appendix B

ANOVA, Tukey’s pairwise comparison tests

**Table:**

Variables	Classification of Universities	Mean Difference		
		Research University	Applied University	General University
IA	Research University	–		
	Applied University	−1.925	–	–
	Comprehensive University	−2.690 *	−0.765	–
MI	Research University	-		–
	Comprehensive University	−5.806 *	–	–
	Comprehensive University	−8.195 *	−2.389 *	–
PsyCap	Research University	–	–	–
	Applied University	1.569	–	–
	Comprehensive University	−8.647 *	−10.217 *	–
MP	Research University	–	–	–
	Applied University	0.061	–	–
	Comprehensive University	−4.274 *	−4.335 *	–

*Note.* * The mean difference is significant at the 0.05 level; IA (internet addiction), PsyCap (psychological capital), MI (mindfulness), MP (moral potency).
